# Molecular cytogenetic analysis and the establishment of a cell culture in the fish species *Hollandichthys multifasciatus* (Eigenmann & Norris, 1900) (Characiformes, Characidae)

**DOI:** 10.1590/1678-4685-GMB-2020-0260

**Published:** 2021-04-16

**Authors:** Letícia Batista Soares, Fabilene Gomes Paim, Lucas Peres Ramos, Fausto Foresti, Claudio Oliveira

**Affiliations:** 1 Universidade Estadual Paulista “Júlio de Mesquita Filho”, Instituto de Biociências, Laboratório de Biologia e Genética de Peixes, Botucatu, SP, Brazil.

**Keywords:** Neotropical fish, cell culture, chromosomes, FISH, repetitive DNAs

## Abstract

*Hollandichthys* is a fish genus of the family Characidae that was until recently considered to be monotypic, with cytogenetic, morphological, and molecular data being restricted to a few local populations. In the present study, the karyotype of a population of *Hollandichthys multifasciatus* was analyzed using classical and molecular cytogenetic approaches for the investigation of potential markers that could provide new perspectives on the cytotaxonomy. *H. multifasciatus* presented a diploid number of 2n=50 chromosomes and a karyotype formula of 8m+10sm+32st. A single pair of chromosomes presented Ag-NORs signals, which coincided with the 18S rDNA sites visualized by FISH, whilst the 5S rDNA sequences were mapped in two chromosome pairs. The distribution of the U snRNA genes was mapped on the *Hollandichthys* chromosomes for the first time, with the probes revealing the presence of the U1 snDNA on the chromosomes of pair 20, U2 on pairs 6 and 19, U4 on pair 16, and U6 on the chromosomes of pair 11. The results of the present study indicated karyotypic differences in comparison with the other populations of *H. multifasciatus* studied previously, reinforcing the need for further research to identify isolated populations or the potential existence of cryptic *Hollandichthys* species.

## Introduction

Characidae (Teleostei, Characiformes) is the most diverse family of Neotropical fishes, with approximately 1,200 small-bodied species (Fricke *et al*., 2020; Malabarba and Malabarba, 2020). *Hollandichthys* Eigenmann, 1909 is a Characidae genus found in coastal rivers from Rio de Janeiro to Rio Grande do Sul with only two species described, *H. multifasciatus* (Eigenmann and Norris, 1900) and *H. taramandahy* Bertaco and Malabarba, 2013 (Lima et al., 2003; Bertaco and Malabarba, 2013). The species of this genus are easily recognized by the presence of longitudinal black stripes (Bertaco and Malabarba, 2013). However, the morphological and molecular data indicate the existence of two or more additional species, reinforcing the need for a thorough taxonomic review of the group to validate and characterize the taxonomic units that may exist in the *H. multifasciatus* complex (Thomaz et al., 2010; Bertaco and Malabarba, 2013).

Cytogenetics has provided an excellent tool for taxonomic analyses, facilitating the identification of species and clarifying the phylogenetic relationships among taxa (Machado *et al*., 2018; Nirchio et al., 2018, 2019). Numerous studies have used chromosomal markers to resolve taxonomic problems (Nakayama et al., 2012) or to distinguish morphologically similar or cryptic species (Centofante et al., 2003; Soto et al., 2018). Genomic evolution can be identified at the level of chromosomal segments, with the mechanisms of insertion, translocation, inversion, and breakage providing important insights into the speciation process (Reiseberg, 2001; Ortiz-Barrientos et al., 2016; Nascimento et al., 2018).

Although cytogenetic data can provide useful insights for the elucidation of the taxonomic status and evolutionary relationships among fish groups that have a complex evolutionary history, and may even permit the identification of cryptic species, cytogenetic data on *Hollandichthys* are still scarce (Table 1). The data available for local populations of the *H. multifasciatus* complex indicate that, despite a conserved diploid number of 2n=50 chromosomes, there are differences among the macro- and microstructure of their karyotypes. Kavalco et al. (2009) concluded that the cytogenetic differences observed in some Neotropical fish groups reflect the existence of cryptic species or, in some cases, a complex of species.

**Table 1 -:** Summary of the karyotypes found in the different samples of *Hollandichthys multifasciatus.*

Species	River/State/Country	2n	FN	Karyotypic formula	Ag-NORs	Reference
*Hollandichthys multifaciatus*	Grande river, Paranapiacaba, São Paulo, Brazil	50	100	10m+12sm+28st	4m; q; i	Carvalho *et al*., 2002
	Antonina	50	100	14m + 18sm + 18st	5m; q; pc	Balen *et al*., 2013
	Guaraqueçaba	50	100	14m + 20sm + 16st	5m; p; pc	Balen *et al*., 2013
	Iguapé River, São Paulo, Brazil	50	100	8m+10sm+32st	4m; q; i	Present study

2n = number diploide; FN = fundamental number; m = metacentric; sm = submetacentric; st= subtelocentric; a= acrocentric; p = short arm; q= long arm; t= terminal; i= interstitial; pc= pericentromeric

The technology of cell culture, very widespread in several areas of Biology, is still little explored in fish cytogenetics, despite being an excellent alternative to obtain quality chromosomal preparations. This little use is mainly related to the difficulty in standardizing the isolation and maintenance techniques of these cell cultures (Amemiya et al., 1984; Bejar et al., 1997; Zhang et al., 1998; Wang et al., 2012; Paim et al., 2018). However, Romanenko et al. (2015) used the culture technique to study chromosomes of the species *Acipenser ruthenus*, a large species, widely used in aquaculture for the production of caviar, which is on the IUCN list of endangered fish. According to the authors, cytogenetic investigations in species such as sturgeon are complicated by the large number of chromosomes, approximately 2n = 120 chromosomes, and the culture allowed to obtain metaphases with high resolution chromosomes so that cytogenetic techniques could be successfully applied and thus enable the study of polyploidy events in the genome of this species, identifying important cytogenetic markers for the characterization of chromosomes.

Although the culture of cells for obtaining chromosomes is not widely used in fish, they have numerous advantages such as the possibility of establishing cryopreserved cell banks, available at any time and, thus, in case of repetition of cytogenetic methodologies or new experiments, it is not necessary to go back to the field in search of new specimens. In this study, we established a cell culture of *H. multifasciatus,* for the first time, to obtain mitotic chromosomes and study its chromosomal characteristics to provide subsidies for comparative analyzes within *Hollandichthys*. We performed a detailed analysis of a new unexplored population of this species, applying conventional and advanced techniques of molecular cytogenetics, that is, Fluorescence in Situ Hybridization (FISH) using six multigenic families (18S and 5S rDNA and U1, U2, U4 and U6 snDNA) and telomeric sequences (TTAGGG)n as probes.

## Material and Methods

Six individuals (one female, three males, and two individuals of undetermined sex) of *Hollandichthys multifasciatus* were collected from a tributary of the Ribeira de Iguape River in Iguape, São Paulo, Brazil (24°42’57.8” S, 47°41’28.3” W). Tissue samples were obtained from these individuals to establish cell cultures. The capture of the individuals was authorized by ICMBio/SISBIO (License number 13843-2). The voucher specimens were deposited in the fish collection of the Laboratory of Fish Biology and Genetics (LBP) at UNESP, Botucatu (São Paulo, Brazil) under catalog number LBP 28762. All procedures were conducted in accordance with the 1001-CEUA protocol of the UNESP-Botucatu Ethics Committee of the Biosciences Institute.

The fish were anesthetized on ice (-2 ^o^C) and the tissue fragments were removed and washed in 0.4% sodium hypochlorite for 30 seconds, 70% ethylic alcohol for 30 seconds each, and then for one minute in Hank’s balanced salt solution (HBSS; ThermoFisher Scientific, Waltham, MA, USA) supplemented with antibiotics (100 U/mL of penicillin and 100 μg/mL of streptomycin; ThermoFisher Scientific, Waltham, MA, USA) and antimycotics (2.5 µg/mL of amphotericin B; ThermoFisher Scientific, Waltham, MA, USA).

The cells were isolated and cultivated according to Paim et al. (2018). For this, tissue fragments were digested with a collagenase solution at 0.003 mg/mL (Millipore-Sigma, Burlington, MA, USA) for 60 min at 28 °C and then centrifuged at 1000 rpm. The cells were cultured in 6- or 12-well plates at 27 °C with 5% CO_2_, and observed daily under an inverted microscope (LEICA DMI 4000B, Leica Microsystems, Wetzlar, Germany), with the culture medium being changed every two days after cell adhesion. When the cells occupied the entire surface of the flasks, they were subcultured in new flasks at a ratio of 1:2. The cells were raised until the fifth step and the resulting cells were retrieved for the chromosomal preparation.

Mitotic chromosomes were prepared using cells from the first to the fifth step of the cell cultures according to Paim et al. (2018). When the cultures presented a high proportion of dividing cells, a colchicine solution (0.0016%) was added to the flasks for 100 min. The medium was then discarded, and the cells were trypsinized and centrifuged. The resulting pellet was resuspended in a hypotonic solution (0.075M of KCl) for 20 min at 37 °C and then fixed in 3/1 methanol and acetic acid. The cell suspension was dropped onto slides and the chromosomes were stained with 5% Giemsa, pH 7,0, for 8 min. Metaphases were photographed under an optical photomicroscope (Olympus BX61) using the cellSens V2.3 software (Olympus), and the images were edited using Adobe Photoshop CS4 Version 6.2. The karyotypes were classified according to Levan et al. (1964), with the chromosomes being identified as metacentric (m), submetacentric (sm), subtelocentric (st), and acrocentric (a).

C-positive heterochromatic bands were identified using the technique described by Sumner (1972), with some adaptations. The chromosomes were stained with propidium iodide according to Lui et al. (2012), and visualized under an optical fluorescence photomicroscope (Olympus BX61) using the cellSens V2.3 software (Olympus). The nucleolus organizer regions (NOR) were identified by the silver nitrate impregnation procedure described by Howell and Black (1980).

Genomic DNA was extracted from muscle tissue using the Wizard Genomic DNA Purification kit (Promega) according to the manufacturer’s instructions. The 18S rDNA, 5S rDNA, U1, U2, U4, U6 snDNA and [TTAGGG]n telomeric sequence probes were obtained by Polymerase Chain Reaction (PCR) using the primers described by Utsunomia et al. (2016), Pendás et al. (1995), Silva et al. (2015), Colgan et al. (1998) and Ijdo et al. (1991). These probes were labeled by PCR using biotin-16-dUTP (Roche Applied Science) for rDNA 18S and snRNA U1 and U4, and digoxigenin-11-dUTP (Roche Applied Science) for the rDNA 5S, snRNA U2 and U6 probes, and the telomeric sequences.

A high stringency was used in the FISH assays, following the protocol of Pinkel et al. (1986), with some adaptations. The slides were incubated in RNase (4% RNAse/2x2SSC) for 1 h at 37 °C, and then fixed in 1% formaldehyde (1x PBS/50mM MgCl_2_) for 10 min at room temperature. The chromosomal DNA was denatured in 70% formamide at 70 °C for 3 min and dehydrated in an alcoholic series of 70%, 85% and 100% for 3 min each. The hybridization solutions containing the probes (10% Dextran sulfate, 50% formamide, 2xSSC, water and 3-4 µL of each probe) were heated to 98 °C for 10 min and the metaphasic chromosomes were incubated with 30 μL of the mix overnight. After hybridization, the slides were washed in 15% formamide for 20 min at 42 °C and for 15 min in 0.1xSSC at 60 °C. After washing, the slides were incubated in 5% NFDM/ 4%xSSC for 15 min at room temperature. The probe signals in the chromosome preparations were detected using antibodies (avidin-FITC and antidigoxy-rhodamine). After, the slides were washed in Tween 0,5%/4xSSC for 5 min at room temperature and dehydrated again in an ethanol series, 3 min each. The chromosomes were counterstained with 4’,6-diamidino-2-phenylindole/antifade (Vector Laboratories), visualized and photographed under an optical fluorescence photomicroscope (Olympus BX61).

## Results and Discussion

**Figure 1 -:**
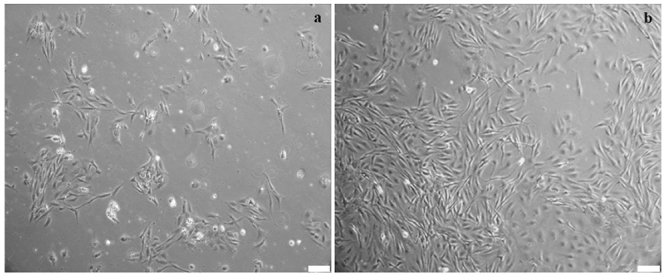
Primary cell culture following the enzymatic digestion of the fin tissue of *Hollandichthys multifasciatus* and the cell confluence after four days **(a)**. Cell population in the second cultivation (**b**). Scale bar = 100 µm.

The cells in the primary cultures of *H. multifasciatus* attained confluence in 2-6 days (Figure 1a), and after subcultivation, they occupied the entire surface of the flasks in 2-4 days (Figure 1b). All the cell lines were maintained and propagated for up to sixth passage. This is the first time that cell cultures have been established for *H. multifasciatus.* Cell culture for *Astyanax* species reported results similar to our studies, with cells with fibroblast morphology and cell confluence in 10 days in primary culture and between two and four days after subcultivation (Paim et al., 2018).

**Figure 2 -:**
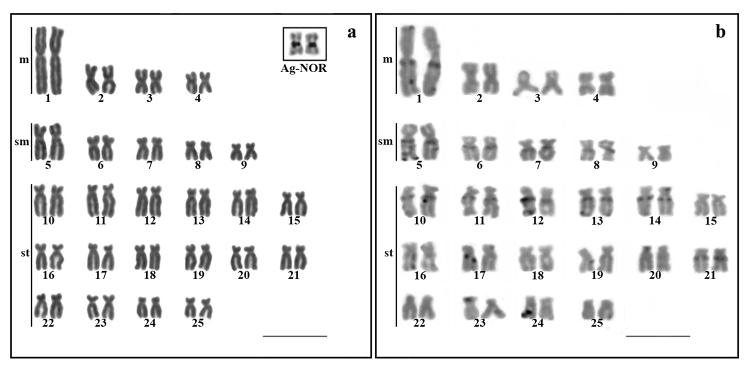
Karyotype of *Hollandichthys multifasciatus* (2n=50 chromosomes) analyzed in the present study by conventional Giemsa staining **(a).** The nucleolus organizing regions are located in the interstitial region of the long arms of chromosomes pair 4 (box). **(b)** C-banded karyotype. Scale bar 10 µm.

The diploid number of the *H. multifasciatus* cells was 2n=50 chromosomes, with a karyotypic formula of 8m+10sm+32st and a fundamental number of 100, in individuals of both sexes (Figure 2a). The NORs were located in the interstitial region on the long arms of the fourth metacentric pair (Figure 2a-box). Blocks of constitutive heterochromatin were visualized in the pericentromeric and interstitial regions of almost all chromosomes. Pairs 1 and 3 also presented small blocks in the terminal regions of the long arms. In the large metacentric pair and pair 16, the heterochromatin was also evident in the terminal region of the short arms (Figure 2b).

**Figure 3 -:**
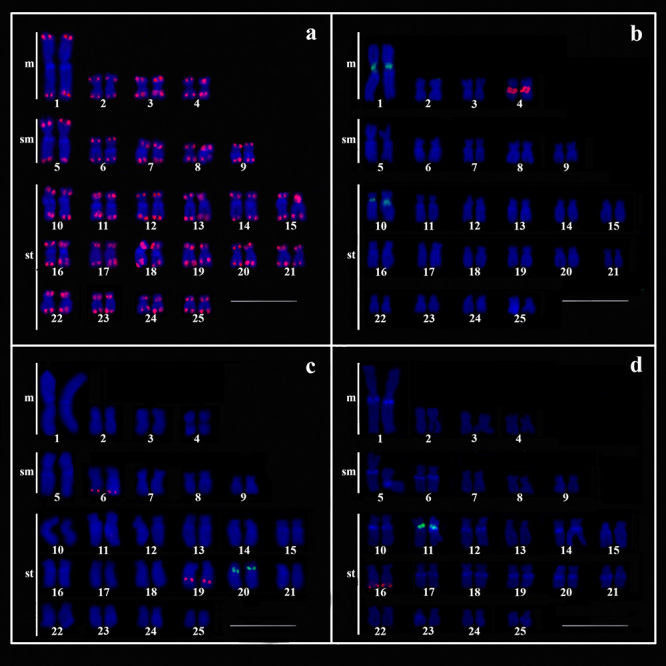
Karyotypes of males of *Hollandichthys multifasciatus* arranged after FISH mapping of (TTAGGG)n sequences (red signals) **(a)**; **(b)** dual-color FISH with 5S (green signals) and 18S rDNA (red signals) sequences; **(c)** dual-color FISH with U1 (green signals) and U2 snDNA (red signals) probes and **(d)** dual-color FISH with U6 (green signals) and U4 snDNA (red signals) probes. The chromosomes were counterstained with DAPI (blue). Scale bar = 10 µm.

The telomeric FISH probe labeled the terminal portion of both arms of all chromosomes, although no interstitial telomeric sequences were observed (Figure 3a). The 18S rDNA sites coincided with the NORs observed by silver staining in the pericentromeric region of the chromosomes of pair 4, whereas the 5S rDNA sites were visualized in the pericentromeric regions of pairs 1 and 10 (Figure 3b). The U1 snDNA genes were located in the terminal region of the short arms of the chromosomes of pair 20, while the U2 probes were located interstitially in the long arms of the chromosomes of pair 6 and the terminal portion of the chromosomes in pair 19 (Figure 3c). The U4 sequences were mapped in pair 16, and the U6 sequences in pair 11 (Figure 3d). The distribution of the snDNA sites in the chromosomes indicate that, while the U4 sites were located in the terminal region, the U1 and U6 sites are located in the interstitial region of the long arms.

The diploid number found here in the *H. multifasciatus* cells was the same as that described previously for this species (Carvalho et al., 2002; Balen et al., 2013), and also for other characids (Duarte et al., 2018; Soto et al., 2018). This diploid number is thus considered to be the plesiomorphic condition in the Characidae (Oliveira et al., 1988). However, the differences observed in the karyotype formula, in comparison with the populations previously analyzed (Table 1) indicate the occurrence of chromosomal rearrangements, such as pericentric inversions and non-Robertson rearrangements, which have likely played a fundamental role in the diversification of the karyotypes of *Hollandichthys* (Balen *et al*., 2013; Soto *et al*., 2018). This variation is especially clear when the karyotypes described by Carvalho *et al.* (2002) and Balen *et al.* (2013) are compared with the one described in the present study. Interestingly, the diploid number remained conserved, despite the presence of marked structural differences in the karyotypes, which reflects the role of rearrangements in this genus. An interesting feature of the karyotype of *H. multifasciatus* is the presence of a large metacentric pair, a common characteristic found in characid species, such as *Hasemania crenuchoides* (Soto *et al*., 2018), *Astyanax janeiroensis* (Carvalho *et al*., 2002), and *Hyphessobrycon reticulatus* (Carvalho *et al*., 2002), which is considered to be an apomorphy and a diagnostic feature of characid clade C (Oliveira *et al*., 2011; Sanchez-Romero et al., 2015).

The distribution of telomeric sequences in the chromosomes provides important insights for the understanding of the evolutionary history of groups or organisms and may indicate the occurrence of chromosomal rearrangements (Ashley and Ward, 1993; Scacchetti et al., 2015). In many fish species, specific sequences revealed by FISH also indicate the presence of interstitial telomeric sites (ITSs) (reviewed in Ocalewicz, 2013; Ribeiro et al., 2014; Scacchetti *et al.,* 2015; Nirchio et al., 2020). Homogeneous signals were observed in the terminal regions of both arms of the chromosomes of *H. multifasciatus*, and no evidence of the presence of ITSs was found, reinforcing the conserved diploid number of this species. However, it is not entirely impossible that the ITSs were lost during speciation process.

The findings of the present study on the distribution pattern of the constitutive heterochromatin in *H. multifasciatus* are consistent with the available data (Carvalho et al., 2002; Balen et al., 2013), including the presence of interstitial blocks, which is likely an ancestral characteristic in the genus. In the present study, however, small blocks of heterochromatin were observed in terminal region of the chromosomes, a feature distinct from the karyotypes described previously (Balen *et al*., 2013), which hints at a specific feature of the Iguape' population. 

Simple Ag-NOR signals are considered to represent a plesiomorphic condition among fishes (Amemiya and Gold, 1998) and have been documented in 72% of teleost species (Gornung, 2013). In the present study, the number and location of the NOR sites are consistent with those described previously in *H. multifasciatus* (Balen et al., 2013), and were confirmed by the location of the 18S rDNA sequences of the pericentromeric region of the chromosomes of pair 4. 

A range of different genes have been mapped in fish species to better understand the evolutionary dynamics of these elements in their genomes (Paim et al., 2017; Santos et al., 2017; Reviewed in Symonová and Howell, 2018; Sochorová et al., 2018; Nirchio et al., 2018). The available data on the mapping of repetitive sequences in *Hollandichthys* was limited to 5S and 18S rDNA sequences. In the present study, the results of the FISH using the 18S rDNA probe, which was confirmed by silver nitrate impregnation, revealed an identical pattern to that observed in the two *H. multifasciatus* populations analyzed by Balen et al. (2013). Even so, the 5S rDNA sites varied in their number and location in the karyotypes of the different populations, with two chromosome pairs being marked in the present study and the population from Antonina (Paraná), whereas signals were observed on three pairs in the population in Guaraqueçaba (Paraná). Similar variation in the number of sites has been documented in a number of other characin species (Silva et al., 2015; Soto et al., 2018; Piscor *et al*., 2020) and have been interpreted as evidence of either sequence dispersion events or the presence of pseudogenes (Barman et al., 2016). The presence of the sequences in the first metacentric pair in the populations studied by Balen *et al.* (2013) and in the present study may be considered an exclusive diagnostic marker for *H. multifasciatus*.

The U snDNA genes (U1, U2, U4, and U6) are conserved in the eukaryotic genome, and are associated with essential mechanisms for the processing of mRNA by associated proteins (Marz et al., 2008). However, few studies are available on the chromosomal organization and dynamics of these genes in fishes (Silva et al., 2015; Santos et al., 2017; Yano et al., 2017; Nirchio et al., 2018; Piscor et al., 2018). The present study provides the first chromosomal mapping of the U genes in *H. multifasciatus*, although they indicated a similar configuration to that found in other characids (Silva *et al*., 2015). The U1, U4 e U6 snDNA sequences occurred in a single chromosome pair, while U2 snDNA was mapped in two pairs. The presence of two chromosomal pairs with U2 snDNA sequences was described for species of *Astyanax* (Silva *et al.,* 2015) and *Triportheus* (Yano *et al.,* 2017) and in other groups a single pair was observed (Piscor *et al*., 2018; Nirchio *et al*., 2020).

As the taxonomy of the genus *Hollandichthys* is incipient, and its evolutionary relationships are still poorly known, cytotaxonomic studies can provide important insights into the processes of differentiation and speciation that characterize its evolutionary history. The results of the present study provide important insights into the macro- and micro-variation identified in the karyotype of *H. multifasciatus* populations analyzed to date, which indicate that this species may have undergone evolutionary shifts during the formation of the Serra do Mar highlands, which isolated the populations in their local river basins (Thomaz et al., 2015). This isolation process is evidenced by the differences in the karyotypes found among the local populations studied to date and reinforces the need for further morphological and genetic analyses to better clarify the potential taxonomic differentiation among the populations. These analyses will be necessary to determine whether the karyotypic differences found to date reflect simple variation in the karyotypes among the populations of a species with an ample distribution, the existence of cryptic species or the existence of a species complex that has yet to be resolved taxonomically.
